# Characterization of clinical and genetic spectrum of Chinese patients with cystic fibrosis

**DOI:** 10.1186/s13023-020-01393-w

**Published:** 2020-06-15

**Authors:** Keqiang Liu, Wenshuai Xu, Meng Xiao, Xinyue Zhao, Chun Bian, Qianli Zhang, Jiaxing Song, Keqi Chen, Xinlun Tian, Yaping Liu, Kai-Feng Xu, Xue Zhang

**Affiliations:** 1 McKusick-Zhang Center for Genetic Medicine, State Key Laboratory of Medical Molecular Biology, Institute of Basic Medical Sciences, Chinese Academy of Medical Sciences & Peking Union Medical College, Beijing, 100005 China; 2 Department of Pulmonary and Critical Care Medicine, Peking Union Medical College Hospital, Chinese Academy of Medical Sciences & Peking Union Medical College, Beijing, 100730 China; 3 Department of Internal Medicine, Peking Union Medical College Hospital, Chinese Academy of Medical Sciences & Peking Union Medical College, Beijing, 100730 China

**Keywords:** Cystic fibrosis, *CFTR*, Chinese, Genetics, Phenotype

## Abstract

**Background:**

Cystic fibrosis (CF) is a rare autosomal recessive disorder caused by biallelic mutations in the cystic fibrosis transmembrane conductance regulator (*CFTR*) gene. The clinical features and mutation spectrum of CF have been well characterized in Caucasians, while limited studies were conducted in Chinese patients.

**Subjects and methods:**

A total of 20 individuals from 19 families were diagnosed with CF in this study. We analyzed the clinical features and screened all coding exons of *CFTR* using a combination of Sanger sequencing and multiplex ligation-dependent probe amplification analysis.

**Results:**

The median age at onset was 9.3 years in our cohort, while the median age at diagnosis was 19 years. The respiratory system was most frequently affected in this study: all patients (100%, 19/19) presented diffuse bronchiectasis and 61.1% (11/18) of patients showed a forced expiratory volume in 1 s below 80% predicted. Six patients (6/20, 30%) exhibited allergic bronchopulmonary aspergillosis (ABPA). Only 4 (4/20, 20%) patients presented pancreatic exocrine insufficiency (PI). Three adult male patients receiving examinations for congenital bilateral absence of the vas deferens were all found positive for the condition. A total of 22 distinct mutations were detected in this cohort, with the variant p.G970D as the most common variant (12/38 alleles, 31.6%). Four variants (p.Y109D, p.I203F, p.D572E, and exon 2–3 deletion) were novel, which expanded the mutation spectrum of Chinese CF patients.

**Conclusions:**

Chinese CF patients showed different clinical features and a distinct *CFTR* mutation spectrum compared with Caucasians. There is a significant diagnosis delay, suggesting the current underdiagnosis of CF in China.

## Background

Cystic fibrosis (CF, OMIM # 219700) is a rare autosomal recessive disorder involving multiple organs, including the respiratory tract, exocrine pancreas, male reproductive system, and exocrine sweat glands. CF is caused by biallelic pathogenic mutations in the cystic fibrosis transmembrane conductance regulator gene (*CFTR*), which is mostly expressed in epithelial cells functioning as a chloride channel protein. CF is mostly reported in Caucasians. According to the 2017 CF Foundation Patient Registry Annual Data Report (available at https://www.cff.org/Research/-Researcher-Resources/Patient-Registry/), there were approximately 30,000 CF patients in the US, with about 1000 newly identified individuals every year. The clinical manifestations and mutation spectrum have been well characterized in Caucasians. By comparison, only approximately 70 Chinese patients have been reported in the literature [1]. It was believed that CF is extremely rare in the Chinese population; however, more than half of these Chinese patients have been reported in the most recent four or five years, [2–6] suggesting that there might be a significant under-estimation of CF incidence in China. With all available data from these studies, we recently made a preliminary summary of the phenotype and genotype spectra of CF in China [1]. Limited data showed that Chinese CF patients have a disposition to present atypical symptoms, mainly displaying pulmonary manifestations with fewer digestive symptoms, and showed a different *CFTR* mutation spectrum. Further studies are warranted to support these findings. In the present study, we collected detailed clinical data and screened *CFTR* mutations in 20 additional Chinese patients to describe the phenotype more accurately and expand the mutation spectrum.

## Methods

### Subjects

From March 2015 to August 2019, patients with suspected CF visiting Peking Union Medical College Hospital (PUMCH) were enrolled in this study. A total of 20 individuals from 19 families were diagnosed with CF according to the 2017 consensus guidelines for CF diagnosis: 1) sweat chloride values ≥60 mmol/L or 2) sweat chloride values in the intermediate range (30–59 mmol/L) in the presence of 2 CF-causing *CFTR* mutations or CFTR dysfunction approved by CFTR physiologic testing; however, 3) individuals with clinical features that may be consistent with CF who have a sweat chloride < 30 mmol/L are less likely to have CF [7]. Informed consent was obtained from all the participants or their parents. All methods carried out in this study were approved by the Institutional Review Board committee at PUMCH.

### Sweat chloride tests

Sweat chloride tests were conducted following a previously described protocol [2]. Briefly, both upper limbs were pre-cleaned for sweat collection. The current was gradually set to 4 mA and maintained for 5 min; meanwhile, 0.5% pilocarpine nitrate and 0.05 mmol/L magnesium sulfate were used in iontophoresis to stimulate sweat. Pre-cleaned dry sterile gauze covered with waterproof surgical tape was used to collect sweat for 30 min. Collected sweat was weighed, and sweat [Na−], [Cl−] and [K−] were measured in triplicate using a chemistry analyzer (A&T EA07 Electrolyte analyzer, A&T Corporation, Japan). Sweat chloride tests were performed at least twice for each patient. For samples with sweat chloride < 60 mmol/L, the value difference between the two tests was required to be < 10 mmol/L; for those with sweat chloride ≥60 mmol/L, the difference threshold was set at < 15 mmol/L. Repeated tests were required for patients with sweat chloride test differences exceeding the above criteria. If all requirements were met, the lower value of multiple tests was used as the input data.

### Pulmonary function tests and nutritional status assessments

Standard pulmonary function was tested by spirometry, and values of forced expiratory volume in the first second (FEV_1_) were expressed as percentages of reference values for South East Asian individuals, as reported by the European Respiratory Society Global Lung Function Initiative, which were adjusted for age, sex, and height [8]. Nutrition outcomes were evaluated by weight/height and body mass index (BMI). For adult patients (> 18 years old), BMI below 18.5 was considered underweight; for children and adolescents under 18, BMI was compared to the BMI growth curves for Chinese children and adolescents aged 0 to 18 years (Table 1) [9].

**Table 1:** Clinical manifestations and *CFTR* mutations for CF patients from this study

No.	Sex	Age at Dx (y)	Age at onset (y)	Weight/ Height (kg/m)	BMI Nutrition status	Family history	Sweat test Cl^−^, mmol/l)	Pulmonary function FEV1% pred; FEV1/FVC)	Pancreatic insufficiency	Sudan III staining	Gastrointestinal symptoms	Other comorbidities	Diagnosis before CF confirmation	Sputum culture	*CFTR* Variant 1		*CFTR* Variant 2	
															cDNA name	Protein name (Legacy name)	cDNA name	Protein name (Legacy name)
1	F	22	10	48/1.62	18.3 underweight	none	113.7	86.4%; 80.60%	none	NA	chronic gastritis	NP	bronchiectasis; TB	*PA*	c.2909G > A ^b^	p.G970D (G970D)	c.3068 T > G	p.I1023R
2	F	11	new born	39/1.5	17.3	none	164.7	85.9%; 74.66%	none	negative	none	ABPA	none	*PA*	c.2909G > A ^b^	p.G970D (G970D)	c.2997_3000delAATT	p.I1000* (3129del4)
3	F	14	12	43/1.65	15.8 BMI < *P*_*15*_^c^	sister died at 7 m for unknown reason	183	60.5%; 62.21%	PI	positive	diarrhea	ABPA	bronchiectasis	*MRSA,* *MSSA*	c.2909G > A ^b^	p.G970D (G970D)	c.2909G > A ^b^	p.G970D (G970D)
4	F	18	8	52/1.62	19.8	None	199	46.7%; 56.87%	none	negative	diarrhea	ABPA	none	*PA, MA*	c.1766 + 5G > T ^b^	(1898 + 5G- > A)	c.1766 + 5G > T ^b^	(1898 + 5G- > A)
5	M	22	2	65/1.7	22.5	None	151.4	61.1%; 61.83%	none	negative	none	none	pneumonia	*PA*	c.607A > T ^a^	p.I203F	c.3635delT	p.V1212Afs*16
6–1	F	30	15	45/1.56	18.5	Sister (6–2): CF	117.9	38.4%; 60.32%	none	negative	none	sinusitis, HA	none	*PA*	c.2909G > A ^b^	p.G970D (G970D)	c.2909G > A ^b^	p.G970D (G970D)
6–2	F	33	30	50/1.5	22.2	Sister (6–1): CF	161.1	91.6%; 73.32%	none	NA	none	none	none	NA	c.2909G > A ^b^	p.G970D (G970D))	c.2909G > A ^b^	p.G970D (G970D)
7	F	4	0.67	15/1.06	13.3 BMI < *P*_*5*_^c^	None	80	NA	PI	NA	fatty diarrhea	sinusitis	none	*PA*	c.325 T > G ^a^	p.Y109D	c.3196C > T ^b^	p.R1066C (R1066C)
8	F	18	13	55/1.7	19.0	None	85	44.6%; 56.69%	none	negative	none	none	bronchiectasis	*PA, MSSA*	c.293A > G ^b^	p.Q98R (Q98R)	c.2353C > T ^b^	p.R785* (R785X)
9	F	18	11	52.5/1.6	20.5	None	120	49.2%; 75.7%	none	negative	none	sinusitis, HA	DPB	*PA, MRSA*	c.54-?_273 +?del (△E2–3) ^a^	–	not detected	–
10	F	18	5	45/1.64	16.7 underweight	None	218.4	25.9%; 56.26%	none	NA	none	sinusitis, HA	bronchiectasis	negative	c.3883_3886delATTT ^b^	p.I1295Ffs*32	c.2909G > A ^b^	p.G970D (G970D)
11	F	16	0.5	30/1.58	12.0 BMI < *P*_*3*_^c^	None	NA	21.8%; 50.34%	none	NA	None	sinusitis, NP	TB	*PA*	c.2909G > A	p.G970D (G970D)	c.1657C > T ^b^	p.R553* (R553X)
12	M	12	5	31/1.48	14.2 BMI < *P*_*3*_^c^	None	210	84.3%; 74.79%	PI	positive	lipid drops in feces, hepatomegaly	sinusitis	bronchiectasis	*PA*	c.405_406dupAC	p.L136Hfs*18	c.1388G > A	p.G463D
13	M	22	14	77/1.78	24.3	None	192	86.1%; 88.35%	none	negative	none	CBAVD, sinusitis	bronchiectasis	*SA, KP, PA*	c.2125C > T ^b^	p.R709* (R709X)	c.2909G > A ^b^	p.G970D (G970D)
14	M	21	10	50/1.7	17.3 underweight	None	133	46.0%; 60.25%	PI	positive	none	ABPA, CBAVD, sinusitis	TB	*PA, MSSA*	c.2547C > A ^b^	p.Y849* (Y849X)	c.2909G > A ^b^	p.G970D (G970D)
15	F	20	12	54/1.7	18.7	None	63	58.3%; 61.37%	none	negative	none	sinusitis, HA	DPB	*PA*	c.3196C > A	p.R1066S (R1066S)	not detected	–
16	F	16	5	50/1.7	17.3	sister: repeated pulmonary infection and died at 10	154	84.7%; 80.91%	none	negative	none	ABPA, sinusitis	bronchopneumonia	*PA*	c.1679 + 2 T > C	–	c.2658-1G > C ^b^	(2790-1G- > C)
17	F	22	14	45/1.58	18.0	none	81	93.6%; 88.36%	none	NA	none	ABPA	bronchiectasis	negative	c.293A > G ^b^	p.Q98R (Q98R)	c.293A > G ^b^	p.Q98R (Q98R)
18	M	18	6	42.5/1.7	14.7 underweight	none	99	26.2%; 55.40%	suspected PI	NA	abdominal distension	CBAVD	none	*PA*	c.595C > T	p.H199Y (H199Y)	c.2909G > A ^b^	p.G970D (G970D)
19	F	44	6	50/1.62	19.1	none	NA	NA	none	NA	none	none	bronchiectasis	*PA*	c.1716C > A ^a^	p.D572E	c.2909G > A ^b^	p.G970D (G970D)

^a^ Novel mutations

^b^ Variants annotated as CF-causing in the CFTR2 database (http://cftr2.org) by functional tests in cell-based systems

^c^ BMI percentile for Chinese children and adolescents [9]

*ABPA* allergic bronchopulmonary aspergillosis, *BMI* body mass index, *CBAVD* congenital absence of the vas deferens, *DPB* diffusive pan-bronchiolitis, *Dx* diagnosis, *FEV1* forced expiratory volume in 1 s, *FVC* forced vital capacity, *HA* hypoalbuminemia, *MA Mycobacterium avium*, *MSSA* methicillin-sensitive *Staphylococcus aureus*; *MRSA* methicillin-resistant *Staphylococcus aureus*; *NA* not available, *NP* Nasal polyp; *PA Pseudomonas aeruginosa*; *PI* pancreatic insufficiency; *KP Klebsiella pneumoniae*; *SA Staphylococcus aureus*; *TB* tuberculosis

### Pancreatic insufficiency (PI)

Patients with PI often experience growth failure and/or abdominal symptoms, which can result from various factors. Measurement of fecal elastase is the most commonly used objective method to screen for or diagnose PI in CF patients with high sensitivity and specificity. But unfortunately, this method is almost unavailable at most clinical centers in China. Instead, the measurement of fecal fat by Sudan III staining is usually used to indirectly assess pancreatic function. Results were reported as negative/positive. Patients with clinical features, such as poor nutrition status and steatorrhea, and positive fecal Sudan III staining were considered to have PI.

### Allergic bronchopulmonary aspergillosis (ABPA)

The diagnosis of ABPA was made following the diagnostic criteria for the classic case proposed by the CF Foundation Consensus Conference in 2003 [10]: 1) unexplained acute or subacute pulmonary function exacerbation; 2) total serum IgE concentration greater than 1000 IU/mL, unless the patient was receiving corticosteroids; 3) immediate cutaneous reaction to *Aspergillus fumigatus* or serum IgE antibody to *A. fumigatus*; 4) precipitating antibodies to *A. fumigatus* or serum IgG antibody to *A. fumigatus* by in vitro tests; and 5) new or recent abnormalities found via chest radiography (infiltrates or mucus plugging) or chest CT (bronchiectasis) that were not cleared with antibiotics and standard physiotherapy.

### Congenital bilateral absence of the vas deferens (CBAVD)

For male patients over 18 years of age, examinations for CBAVD were recommended. The diagnosis of CBAVD was achieved based on these criteria: the presence of normal to slightly smaller testicles, a non-palpable vas deferens, normal plasma levels of follicle-stimulating hormone, and reduced ejaculate volume (< 1 mL) [11].

### Mutation screening of *CFTR*

Genomic DNA was extracted from the peripheral blood of the patient and their parents if available following standard methods. Direct sequencing for all 27 exons of *CFTR* and flanking sequences was performed as previously described [2]. Sequencing traces were analyzed using the CodonCode Aligner software (CodonCode Aligner Corporation; Centerville, MA, USA), and variant nomenclature was described according to the transcript reference NM_00492.3. Multiplex ligation-dependent probe amplification (MLPA) analysis was conducted to screen for potential gross rearrangements for those with only one or no mutation identified via exon sequencing using a commercial MLPA kit (SALSA® P091-D1 CFTR, MRC-Holland; Amsterdam, The Netherlands). Real-time quantitative PCR (qPCR) of genomic DNA was performed to verify the gross deletion detected by MLPA analysis as described previously, [4] with the qPCR primer pairs CFTR-E2 (F 5′-TGTAAGAGATGAAGCCTGGTATT-3′ and R 5′-AGGCGCTGTCTGTATCCTTT-3′) and CFTR-E3 (F 5′-TGGGATAGAGAGCTGGCTTCA-3′ and R 5′-ACACCTATTCACCAGATTTCGT-3′). Subsequently, the deletion breakpoints were characterized by long-range PCR and Sanger sequencing.

### Genotyping of modifier genes for CF lung disease

A thorough literature review was conducted to summarize the modifier genes associated with CF lung disease. Specific primers were designed (Table S1) for the genotyping of each locus. To construct the associated haplotypes, corresponding loci were also sequenced for the patients.

## Results

In total, 20 patients (15 females and 5 males) from 19 Chinese families diagnosed with CF were recruited into this study. The age at symptom onset ranged from newborn to 30 y with a median of 9.3 y in this cohort. The median age at diagnosis was 19 y, ranging from 4 to 44 y. Patient 3 had a sister who died at 7 months for an unknown reason, and the elder sister of Patient 16 died at 10 y due to repeated pulmonary infection, suggesting a suspected history of CF. Patients 6–1 and 6–2 were sisters both suffering from CF. All patients denied consanguineous marriage in their families.

### Clinical manifestations of CF patients in this cohort

The respiratory tract is the most frequently affected system in this cohort. Almost all patients had diffuse bronchiectasis (100%, 19/19), and 50% (10/20) had sinusitis. Six patients (6/20, 30%) exhibited ABPA. *Pseudomonas aeruginosa* (*PA*) was the most common pathogen observed in our patients with a frequency of 16/19 (84.2%). Only 2 patients showed negative results of sputum culture. Eleven out of 18 (11/18, 61.1%) patients who accepted pulmonary function testing had an FEV_1_ below 80% predicted. There were 4 (4/20, 20%) patients diagnosed with PI, all of whom presented with poor nutritional status. An additional patient (Patient 18) was diagnosed with suspected PI; this individual exhibited abdominal distension and was very severely underweight but without measurement of fecal fat or the observation of fatty diarrhea. All patients showed normal liver function, and none manifested liver diseases or abnormal findings by CT/B-ultrasound scan, except for Patient 12, who presented with hepatomegaly. An important comorbidity of CF was CBAVD [12]. Three adult male patients accepted the CBAVD examination in this cohort, and all of them were diagnosed with CBAVD (3/3, 100%). The other two male patients were too young or were simply reluctant to accept the examination. Sweat chloride testing results were obtained from 18 patients, all of whom showed elevated sweat chloride concentrations (> 60 mmol/L). Detailed information on clinical manifestations of the patients were summarized in Table 1.

### Germline *CFTR* variants detected in this study

**Fig. 1:**
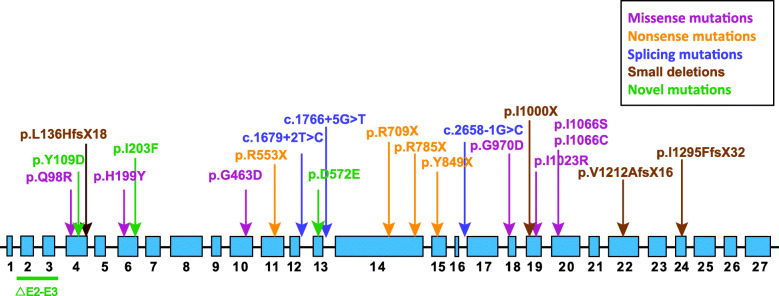
*CFTR* mutations detected in this CF cohort. Different mutation types are shown in the colors indicated in the upper panel; the gross deletion of exons 2–3 is indicated with a green solid line in the lower panel. The novel mutations identified in the current study are highlighted in green

All patients were screened for *CFTR* mutations by direct sequencing and MLPA analysis. Biallelic *CFTR* variants were detected in all patients, except for Patients 9 and 15, for whom only one mutation was detected (Table 1). In total, 22 distinct variants were detected, including 10 missenses, 5 nonsenses, 3 indels, 3 splicing mutations and 1 gross deletion (Fig. 1). Among them, 4 mutations (c.325 T > G, p.Y109D; c.607A > T, p.I203F; c.1716C > A, p.D572E and exon 2–3 deletion) turned out to be novel mutations. In the 19 unrelated probands, the variant c.2909G > A (p.G970D) was the most common variant detected, with an allele frequency of 31.6% (12/38 alleles). The variants c.293A > G (p.Q98R) and c.1766 + 5G > T were also observed more than once. The most frequent mutation for Caucasian CF patients, p.F508del, was not observed in our cohort.

**Fig. 2:**
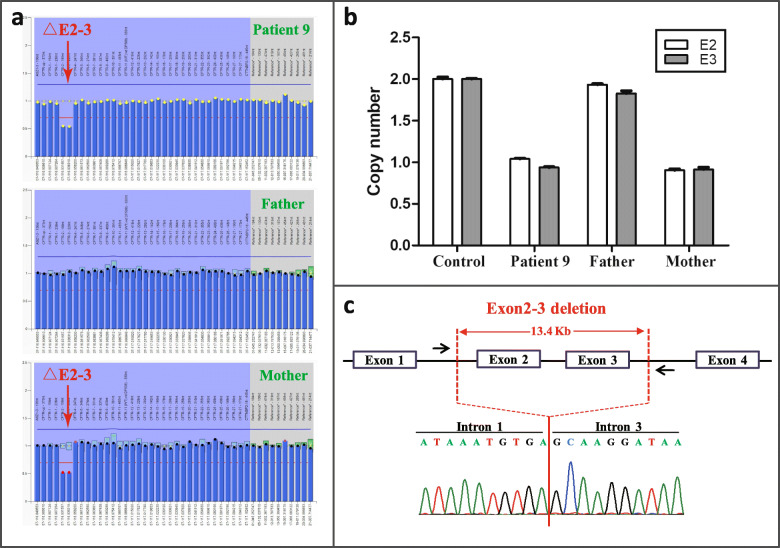
*CFTR* exons 2–3 deletion found in patient 9. **a** Exons 2–3 deletion in Patient 9 and her mother, which was detected by MLPA. The x-axis shows the genomic positions of the probes, and the y-axis represents the signal ratio compared with control. The red arrow represents the heterozygous deletion. **b** Quantitative real-time PCR confirmed the *CFTR* exons 2–3 deletion in the patient, which was inherited from her mother. Experiments were performed in triplicate. **c** Sanger sequencing revealed a deletion of approximately 13.4 Kb encompassing *CFTR* exons 2–3. The breakpoints are shown using red lines

Direct sequencing of all *CFTR* exons failed to identify any pathogenic mutations in Patient 9. Subsequently, MLPA analysis of *CFTR* was performed and revealed a heterozygous gross deletion involving exons 2–3 (c.54-?_273 +?del, △E2–3) inherited from her mother (Fig. 2a). The maternal deletion of *CFTR* exons 2–3 was confirmed by qPCR (Fig. 2b). Subsequent breakpoint characterization showed that there was a deletion of 13.4 Kb encompassing exons 2 and 3 of *CFTR* (Fig. 2c). The complete removal of exons 2–3 resulted in a frameshift and premature termination codon (p.S18Rfs*16). A second mutation from the paternal chromosome was not detected.

### Different phenotypes and modifier gene genotypes of patients 6–1 and 6–2

**Fig. 3:**
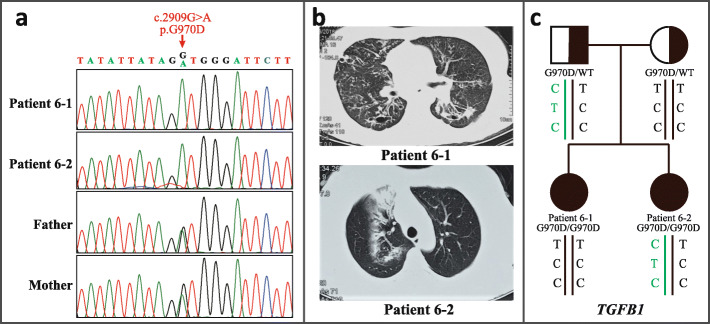
Different lung manifestations and potential modifier loci in Patients 6–1 and 6–2. **a** As siblings, Patients 6–1 and 6–2 are both homozygous for p.G970D, and their parent are both heterozygous carriers. **b** Chest CT showed that Patient 6–1 presented diffuse bronchiectasis in the lungs bilaterally, while Patient 6–2 only had focal bronchiectasis in the right upper lobe. **c** Genotypes of the three SNPs associated with lung disease severity (rs1800469, ‘-509’, rs1982073, ‘codon 10’, and rs8179181, ‘intron 5’) in the *TGFB1* gene. Patient 6–1 carried the risk genotype CC at codon 10, indicated in red, and Patient 6–2 carried the protective C-T-C haplotype, shown in green

Despite bearing the same homozygous p.G970D mutation (Fig. 3a), Patient 6–1 showed more severe lung disease, with diffuse bronchiectasis in bilateral lungs and continuous deterioration of pulmonary function (FEV_1_ = 38.4% Pred, FEV_1_/FVC = 60.32%), compared with her older sister, Patient 6–2, who had a normal daily life, with focal bronchiectasis restricted in the right upper lobe (Fig. 3b) and quite normal pulmonary function (FEV_1_ = 91.6% Pred, FEV_1_/FVC = 73.32%).

Most CF lung disease-associated modifier genes bore the same genotype between the siblings (Table S1). Interestingly, Patient 6–1 carried the risk genotype codon 10 CC (rs1800470) [13] but not the protective haplotype composed of the − 509 C, codon 10 T and intron 5 C allele (C-T-C haplotype) [14] in *TGFB1*, which was opposite for Patient 6–2 (Fig. 3c). Besides, there were three other previously reported lung function-associated SNPs showing different genotypes for the siblings, for which Patient 6–1 carried all the risk alleles (A allele for rs3103933 in *MUC4/MUC20*, [15] C allele for rs9268905 [16] and rs9391781 [15] in *HLA II*), whereas Patient 6–2 did not (Table S1).

## Discussion

Understanding the phenotype and genotype features of a specific population helps to make correct diagnosis of CF patients. Numerous CF studies conducted in Caucasians have successfully characterized the clinical features and *CFTR* mutation spectrum of Caucasian CF patients. In contrast, limited knowledge of the spectrum of CF phenotype and genotype among Chinese individuals is available. Recently, a systematic review preliminarily summarized the clinical and genetic characteristics of 71 reported Chinese CF patients [1]. The authors also reported a significant under-recognition and diagnosis delay of CF in China, with a median age at onset of 1 y and a median age at diagnosis of 10 y [1]. The present study collected 20 CF patients, of which the median ages at onset and diagnosis were 9.3 y and 19 y, respectively. The reason for the much later ages of onset and diagnosis in this cohort might be due to selection bias because all these patients were recruited from the Department of Pulmonary and Critical Care Medicine, a clinic mainly for adults in PUMCH. Despite that, a similar significant diagnosis delay of approximately 10 years was also observed in this study. Out of 20 patients, 15 (15/20, 75%) had experienced misdiagnosis, including bronchiectasis, diffusive pan-bronchiolitis, pulmonary tuberculosis, and pneumonia.

As to clinical manifestation, respiratory symptoms were those most commonly observed in our cohort, consistent with the results of previous studies performed on CF patients of Chinese origin [1, 3, 5]. Interestingly, approximately 30% of patients in this study presented ABPA, which was far more than what has been reported in Caucasians (7–9%) [17]. Similarly, several recent studies [2, 3, 5] also have reported a higher rate of ABPA in Chinese patients, ranging from 37.5 to 57.1%. Furthermore, our recent review demonstrated that out of all 71 reported Chinese CF patients, 15 had developed ABPA, reaching a high rate of 21.1% [1]. Therefore, from the data in these independent studies, we would conclude that Chinese CF patients might be more prone to developing ABPA than Caucasians.

In addition, a lower frequency (4/20, 20%) of PI was found in this cohort. In contrast, the frequency for Caucasians reported in the 2017 Cystic Fibrosis Foundation Patient Registry Annual Data Report (available at https://www.cff.org/Research/Researcher-Resources/Patient-Registry/) was approximately 85%. Similar results can be found in several recent studies conducted in the Chinese population [1–3, 5]. However, what we should notice is that Sudan III staining may produce false-negative results by testing spot stool specimens because fecal fat may not be evenly distributed in individuals consuming a mixed diet. Moreover, about 40% (8/20) of patients refused fecal fat measurement. Thus, we cannot exclude the possibility that PI was under-recognized in these Chinese CF patients. PI and PS status in CF are predisposed by the genotype at the *CFTR* locus [18, 19]. The most common mutation detected in Caucasians, p.F508del, and other mutations severely impairing CFTR function were reported to be associated with PI [18]. Thus, the substantially different *CFTR* mutation spectrum from that of Caucasians (see below) might be responsible, at least partially, for the lower frequency of PI observed in Chinese.

CBAVD is one of the comorbidities commonly seen in Caucasian male CF patients [12]. There was only one adult male Chinese CF patient previously reported, and he was diagnosed with CBAVD [1]. In this study, 3 of the 5 male patients received semen tests and/or urological ultrasound examination, all of whom turned out to have CBAVD. Thus, all 4 Chinese male patients examined, including the 3 reported here, showed manifestation of CBAVD. In other words, it appears that CBAVD is also frequently present in male CF patients of Chinese origin. Further studies with larger sample sizes or long-term follow-up data may make us more confident to draw this conclusion.

Three decades have passed since the cystic fibrosis gene was identified [20]. Extensive efforts have been directed towards research on *CFTR* and this disease. Over 2000 mutations have been detected in CF patients across the world, as recorded in the Cystic Fibrosis Mutation Database (CFTR1 database, http://www.genet.sickkids.on.ca). The mutation spectrum has been well established in Caucasians. A distinct *CFTR* mutation spectrum in Chinese has been suggested in previous studies [1, 2]. In the present study, we detected 22 distinct mutations, with p.G970D as the most common mutation, accounting for 31.6% (12/38 alleles) of *CFTR* alleles. Mutations detected more than once also included two other variants, p.Q98R and c.1766 + 5G > T. The three mutations were all among the five most common mutations observed in Chinese [1]. In contrast, the most frequent mutation in Caucasians, p.F508del, was not observed; only one mutation, p.R553X, detected in Patient 11, was in the screening panel recommended by the American College of Medical Genetics, which consists of the 23 most common mutations in Caucasians [21]. Thus, the present study further confirmed the ethnic-specific mutation spectrum in patients of Chinese origin. Eight of the variants detected in this cohort were not included in the CFTR1 database, which have been uploaded with corresponding clinical data before the submission of this paper.

Four novel variants (p.Y109D, p.D572E, p.I203F, and △E2–3) never reported before were detected in this study. According to the CFTR1 database, the variant p.Y109D caused amino acid changes at the same position as the reported missense mutations p.Y109N and p.Y109C. Similarly, the variant p.D572E led to amino acid changes at position 572, which is the same as reported mutation p.D572H. This is regarded as moderate evidence supporting their pathogenicity, but they cannot be assumed pathogenic [22]. The potential pathogenicity of the variant p.I203F was unknown. Further functional studies for these missense mutations are warranted to determine their effects on CFTR function. The deletion of *CFTR* exons 2–3 found in Patient 9 was the fourth gross rearrangement reported in CF patients of Chinese origin. △E2–3 is very like to be a pathogenic mutation because it was predicted to result in a pre-mature termination codon and no functional CFTR protein. A similar deletion of 21 Kb, also removing exons 2–3, was found to be a frequent and severe CF-causing mutation in Central and Eastern Europe, [23] supporting the pathogenicity of the △E2–3 deletion reported here. It is noteworthy that the other three gross deletions were also detected by our group [2–4]. There might be an underestimated detection rate of rearrangement in *CFTR* in Chinese patients, for which overlooking and the inaccessibility of MLPA analysis should be responsible. Further emphasis should be placed on the necessity of MLPA analysis in routine *CFTR* mutation screening in Chinese patients, especially for those with only one or no mutations identified via direct sequencing of *CFTR* exons.

Interestingly, the siblings (Patients 6–1 and 6–2) carried different genotypes in reported modifier genes (Table S1), which is consistent with previous studies [13, 14] and might contribute to the substantial differences in their lung diseases. However, what we should notice is that these modifiers can only explain a limited proportion of the variability in lung disease, and the reliability of the associations remains to be replicated. Additionally, other factors, such as environmental exposure, may also contribute to the variability in the pulmonary phenotype observed in patients.

There were some limitations in our research. First, all patients were recruited from a single center, the Department of Pulmonary and Critical Care Medicine, PUMCH. Some of the patients came to our center due to suspected ABPA. Therefore, the higher rate of ABPA in Chinese CF patients found in the present study should be carefully used because of potential selection bias. Second, due to cultural reasons, patients often refuse semen examination, making it difficult to screen CBAVD in Chinese CF patients. Studies with more patients accepting CBAVD screening are warranted to confirm the high frequency of CBAVD observed in this study. Third, there are several extremely high sweat chloride values, which are not physiologically possible and may be due to sampling or technical issues [24]. These abnormally high values, although won’t change the final diagnoses, should be used carefully and need to be repeated whenever possible. Moreover, the inaccessibility of standard tests for PI diagnosis in China makes it difficult to assess the true number of CF patients with PI. As an objective method with high accuracy, the measurement of fecal elastase warrants being conducted on Chinese CF patients in the future.

## Conclusion

In summary, the present study showed some different features of the clinical manifestations in Chinese CF patients compared with Caucasians, including greater ABPA presence and a lower frequency of PI. The additional patients with CBAVD reported in this study indicate a probable, similarly high frequency of CBAVD in male CF patients of Chinese origin, which needs further studies to confirm. We also observed a distinct *CFTR* mutation spectrum in Chinese, with p.G970D as the most frequent mutation. Four novel mutations were reported, which expanded the mutation spectrum. There is still a significant diagnosis delay and under-recognition of CF in China. Better characterization of the phenotype and genotype spectra and increasing physician awareness of CF will help to improve the current situation in China.

## Supplementary information

**Additional file 1: Table S1**. Genotypes of modifier genes for CF lung diseases in patients 6–1 and 6–2.

## Data Availability

The datasets supporting the conclusions of this article are included within the article and its additional file.

## Abbreviations

ABPA: Allergic Bronchopulmonary Aspergillosis
BMI: Body mass index
CBAVD: Congenital bilateral absence of the vas deferens
CF: Cystic Fibrosis
*CFTR*: Cystic fibrosis transmembrane conductance regulator
CT: Computed Tomography
FEV_1_: Forced expiratory volume in the first second
FVC: Forced vital capacity
MLPA: Multiplex ligation-dependent probe amplification
PI: Pancreatic Insufficiency
PUMCH: Peking Union Medical College Hospital
qPCR: Real-time quantitative PCR
