# Supplementary material for: Characterization of clinical and genetic spectrum of Chinese patients with cystic fibrosis
Source: Orphanet J Rare Dis. 2020 Jun 15;15:150. doi: 10.1186/s13023-020-01393-w (PMC7294671; doi:10.1186/s13023-020-01393-w)
Supplement: Supplementary file 1 — Additional file 1: Table S1. Genotypes of modifier genes for CF lung diseases in patients 6–1 and 6–2. [file 13023_2020_1393_MOESM1_ESM.docx]

**Table S1. Genotypes of modifier genes for CF lung diseases in patients 6-1 and 6-2.**

| **SNPs** | **Chr.** | **Position** | **Nearest gene** | **Risk allele** | **Genotype** | | | | **Forward primer** | **Reverse primer** | **References** |
| --- | --- | --- | --- | --- | --- | --- | --- | --- | --- | --- | --- |
|  |  |  |  |  | **Patient 6-1** | **Patient 6-2** | **Father** | **Mother** |  |  |  |
| rs12793173 | 11 | 34,812,657 | *EHF/APIP* | C | T/T | T/T |  |  | TTTGTTGGCCGTGGACTTG | CAGCCACTGGTTCTTTGCTT | Wright FA, et al. 2011 [1] |
| rs10742326 | 11 | 34,788,463 |  | A | A/G | A/G |  |  | CTCCCAGTGAAAGGCCTACA | CCACACTCTCAACCCCTACA | Dang H, et al. 2016 [2] |
| rs1403543 | X | 116,170,939 | *AGTR2/SLC6A14* | A | A/G | A/G |  |  | TCTGGTGCTATTACGTCCCA | TGCCTAAACACACTCCTGTAAG | Wright FA, et al. 2011 [1] |
| rs5952223 | X | 116,255,308 |  | T | C/C | C/C |  |  | TGCAGAAAACGCCTTTGACA | CCCTGGCCAAAACTTCCAAA | Corvol H, et al. 2015 [3] |
| rs3788766 | X | 116,435,671 |  | T | T/C | T/C |  |  | CACACATGCACATCAGCCTT | ACTCAGTCTGCCCAGGTAAC | Li W, et al. 2014 [4] |
| rs3103933 | 3 | 195,758,569 | *MUC4/20* | A | G/A | G/G |  |  | TAGGGCTCGGGGTCTCTAAC | AACTGATTCAAGGCTGGGCG | Corvol H, et al. 2015 [3] |
| rs9268905 | 6 | 32,464,300 | *HLA II* | C | G/C | G/G |  |  | CACATGCCTAAACCTCACACA | TGGTCTGTATTAGCGTGGAGT | Wright FA, et al. 2011 [1] |
| rs9391781 | 6 | 32,467,073 |  | C | G/C | G/G |  |  | GGCTCCCAAACTACCTGTGA | TCAGTTCCCAGAACTTCGGT | Corvol H, et al. 2015 [3] |
| rs17563161 | 5 | 497,509 | *SLC9A3* | A | G/G | G/G |  |  | GGGTCTCAAATCCGGGTTCT | AGGGGATCTGGAAGCTTCAG | Li W, et al. 2014 [4] |
| rs57221529 | 5 | 586,509 |  | G | A/A | A/A |  |  | GGGAGGAGGGTGATGAAGTT | CCCTCAAATGGAAGATGCGT | Corvol H, et al. 2015 [3] |
| rs7096206 | 10 | 52,771,925 | *MBL2* | G | G/G | G/G |  |  | TCCTCATATCCCCAGGCAGT | GGGAATCAGCTGCCCAGATA | Gravina LP, et al. 2015 [5] |
| rs5030737 | 10 | [52,771,482](https://genome.ucsc.edu/cgi-bin/hgTracks?hgsid=708275623_tZYZmWAeQZE9a9dIwsbIWoNo5hw1&db=hg38&position=chr10%3A52771482-52771482) |  | C | C/C | C/C |  |  |  |  |  |
| rs1800450 | 10 | [52,771,475](https://genome.ucsc.edu/cgi-bin/hgTracks?hgsid=708275623_tZYZmWAeQZE9a9dIwsbIWoNo5hw1&db=hg38&position=chr10%3A52771475-52771475) |  | G | A/G | A/G |  |  |  |  |  |
| rs1800451 | 10 | [52,771,466](https://genome.ucsc.edu/cgi-bin/hgTracks?hgsid=708275623_tZYZmWAeQZE9a9dIwsbIWoNo5hw1&db=hg38&position=chr10%3A52771466-52771466) |  | G | G/G | G/G |  |  |  |  |  |
| rs7817 | 7 | [112,475,603](https://genome.ucsc.edu/cgi-bin/hgTracks?hgsid=708275623_tZYZmWAeQZE9a9dIwsbIWoNo5hw1&db=hg38&position=chr7%3A112475603-112475603) | *IFRD1* | C | C/T | C/T |  |  | GCAGCCTTCAAAGCTCGAAC | TACAGCAGCTTTTCCAGGGG | Gu Y, et al. 2009 [6] |
| rs1800469 | 19 | [41,354,391](https://genome.ucsc.edu/cgi-bin/hgTracks?hgsid=708275623_tZYZmWAeQZE9a9dIwsbIWoNo5hw1&db=hg38&position=chr19%3A41354391-41354391) | *TGFβ1* | - | T/T | C/T | C/T | T/T | TGGCACAGTGGTCAAGAGC | CAGAGAAAGAGGACCAGGCG | Drumm ML, et al. 2005 [7]  Bremer LA, et al. 2008 [8] |
| rs1800470 | 19 | [41,353,016](https://genome.ucsc.edu/cgi-bin/hgTracks?hgsid=708275623_tZYZmWAeQZE9a9dIwsbIWoNo5hw1&db=hg38&position=chr19%3A41353016-41353016) |  | C | C/C | C/T | C/T | C/C | GACTTTTCCCCAGACCTCGG | CTTCACCAGCTCCATGTCGA |  |
| rs8179181 | 19 | [41,332,301](https://genome.ucsc.edu/cgi-bin/hgTracks?hgsid=708275623_tZYZmWAeQZE9a9dIwsbIWoNo5hw1&db=hg38&position=chr19%3A41332301-41332301) |  | - | C/C | C/C | C/C | C/C | CAGTGGGTGCAAGGGAGAC | TTTCTCCCCATCCTGCCAAC |  |

References:

1. Wright FA, Strug LJ, Doshi VK, Commander CW, Blackman SM, Sun L, et al. Genome-wide association and linkage identify modifier loci of lung disease severity in cystic fibrosis at 11p13 and 20q13.2. Nat Genet. 2011;43(6):539-46.

2. Dang H, Gallins PJ, Pace RG, Guo XL, Stonebraker JR, Corvol H, et al. Novel variation at chr11p13 associated with cystic fibrosis lung disease severity. Hum Genome Var. 2016;3:16020.

3. Corvol H, Blackman SM, Boelle PY, Gallins PJ, Pace RG, Stonebraker JR, et al. Genome-wide association meta-analysis identifies five modifier loci of lung disease severity in cystic fibrosis. Nature communications. 2015;6:8382.

4. Li W, Soave D, Miller MR, Keenan K, Lin F, Gong J, et al. Unraveling the complex genetic model for cystic fibrosis: pleiotropic effects of modifier genes on early cystic fibrosis-related morbidities. Hum Genet. 2014;133(2):151-61.

5. Gravina LP, Crespo C, Giugno H, Sen L, Chertkoff L, Mangano A, et al. Mannose-binding lectin gene as a modifier of the cystic fibrosis phenotype in Argentinean pediatric patients. J Cyst Fibros. 2015;14(1):78-83.

6. Gu Y, Harley IT, Henderson LB, Aronow BJ, Vietor I, Huber LA, et al. Identification of IFRD1 as a modifier gene for cystic fibrosis lung disease. Nature. 2009;458(7241):1039-42.

7. Drumm ML, Konstan MW, Schluchter MD, Handler A, Pace R, Zou F, et al. Genetic modifiers of lung disease in cystic fibrosis. N Engl J Med. 2005;353(14):1443-53.

8. Bremer LA, Blackman SM, Vanscoy LL, McDougal KE, Bowers A, Naughton KM, et al. Interaction between a novel TGFB1 haplotype and CFTR genotype is associated with improved lung function in cystic fibrosis. Human molecular genetics. 2008;17(14):2228-37.
